# Validation of a Method to Measure the T2 Value from the Color Mapping by Hue Value

**DOI:** 10.2174/1573405618666220907110157

**Published:** 2023-05-17

**Authors:** Takehito Hananouchi, Makishi Nakayama

**Affiliations:** 1 Medical Engineering Laboratory, Department of Mechanical Engineering, Faculty of Engineering, Osaka Sangyo University, Daito, Osaka 574-8530, Japan;; 2 Department of Orthopaedic Surgery, Shimada Hospital, 100-1 Kashiyama, Habikino, Osaka, Japan;; 3 Intelligent Control System (Laboratory), Department of Mechanical Engineering, Faculty of Engineering, Osaka Sangyo University, Daito, Osaka 574-8530, Japan

**Keywords:** T2 mapping, color mapping, RGB (Red, Green, Blue), HSV (Hue, Saturation, Value), medical imaging, MRI

## Abstract

***Background*:** Color mapping using quantitative MRI (Magnetic Resonance Imaging) is now being reported in various medical fields to be useful in showing tissue conditions and morphological perspectives. Specifically, T2 mapping, as one of the color mapping has been used to describe cartilage conditions in orthopedics. However, for orthopedic physicians in outpatient clinics, the color mapping shows only the colors on the mapping to patients without explaining their numerical values.

***Methods*:** Our study proposed an approach to measure T2 values based on the hue value converted from Red, Green, Blue information on the processed color map to address this issue. We evaluated the validity of our method with 25 subjects.

***Results*:** Our proposed method showed a good and high correlation coefficient (r = 0.9924, p < 0.0001), and the difference in the T2 values using dedicated software on the console of the MRI scanner and our method was small (its absolute value was approximately 1.5, p = 0.008).

***Conclusion*:** Therefore, we consider the proposed method is an alternative approach to show the T2 value when the color mapping is available.

## 1 INTRODUCTION

**Fig. (1):**
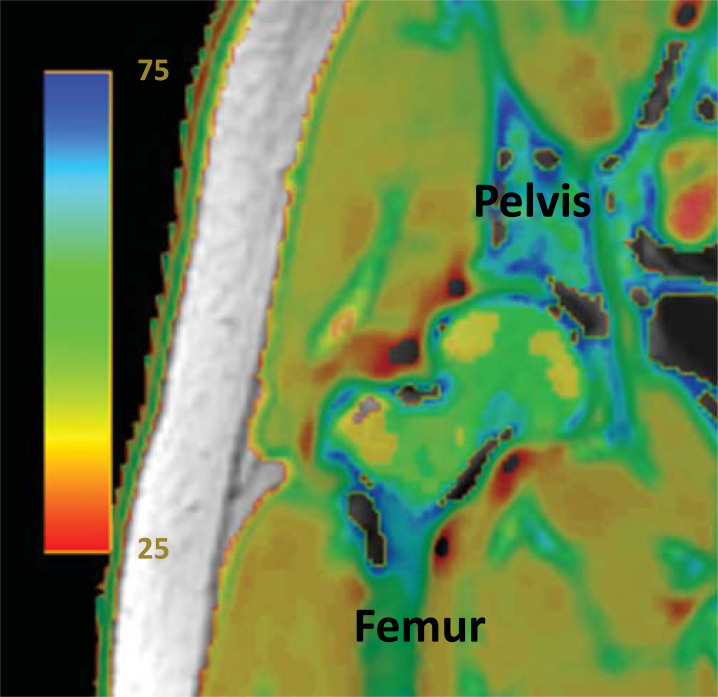
A figure sample of the Hip joint consists of pelvis and femur bones with a color bar of the T2 mapping.

The color mapping system by quantitative MRI, such as T2, T2 star, or T1 rho is now being reported in various medical fields (heart, liver, kidney, brain, musculoskeletal disorders, etc.) to show tissue conditions and morphological perspectives [1-5]. Of the color mapping system, T2 mapping (*i.e*. color mapping by T2 value) has been used to describe cartilage or other soft tissues conditions in the field of orthopedics, e.g., articular cartilage degeneration before morphological cartilage damage can be detected [5-8]. The prepared MRI images of the same scan site with several different TEs, T2 value is originally determined using the time constant of T2 for the decaying enhancement [9]. Then, the T2 color mapping is automatically made by converting one T2 value to correspond to one specific color in a rainbow color (Fig. **1**): One sample of the rainbow color is “Jet”; https://www.mathworks.com/help/matlab/ref/jet.html).) in dedicated software on the console of MRI scanner.

At a glance, the color mapping might be useful in explaining imaging results to patients. However, it is doubtful that the color mappings are really useful to the orthopedic physician in an outpatient clinic as the end user. This is because the physicians cannot measure quantitative values on the color mapping since the image was already processed without knowing the numerical values. The orthopaedic physicians cannot tell patients, “The cartilage condition in this area does not seem to be good because the T2 value is 80 in the area”, but “This area does not seem to be good because the color is relatively blue” with referencing of the color bar on the color mapping image. This is also the same situation when orthopedic physicians see the image media of T2 mapping from external hospital institutions brought by some patients. In this situation, the color mapping images do not have T2 values quantitatively. Additionally, when the difference between normal and diseased areas is relatively small, the orthopaedic physicians may not provide an appropriate diagnosis. In the case of seeing the T2 color mapping by a dedicated software on the console of the MRI scanner at the radiology department, the T2 value can be known. Its area is not only at one pixel but also in any area of ROI (region of interest). This is a MOTTAINAI (one Japanese expression; it can be translated as 'What a waste!' or 'Don't be wasteful!') situation [10].

In order to address this issue, we have been planning to make software or application with the following method to calculate T2 values from the image data of already processed T2 colored maps. Normally, it is known that the color information of RGB (Red, Green, and Blue) styles can be picked up. However, since the number of parameters on the color scale bar of JET is only one and one parameter is changed linearly, a conversion method should be necessary to indicate one value of T2 from RGB information which has three parameters (*i.e*., red, green, and blue). According to a website (https://stackoverflow.com/questions/28685724/ to-categorize-jet-colormap-by-pixels-of-some-colors), we determined to use hue value from HSV (hue, saturation, and value) styles which are converted from RGB styles, because it is changed linearly on the color scale bar of JET. That is, in the case of red at the bottom of the scale bar, the RGB value is 255, 0 and 0. Then, the hue value converted from the RGB value is 0. In another case, which is blue at the top of the scale bar, the RGB is 0, 0 and 255. Then, the hue value converted from the RGB value is 66. Finally, we determined the following formula to detect the T2 value;

T2 value = “hue value” × 50/66 + 25

This method to know the T2 value might substitute the normal method to indicate the T2 value. However, there is no report to validate whether T2 values from color information on the processed image (*i.e*., our proposed method) are substituted for T2 values, which are automatically obtained at the original workstation. Therefore, we determined to investigate it.

The purpose of this study is to validate our proposed method. For this purpose, we compared both of the T2 values, especially their difference and correlation.

## 2 MATERIALS AND METHODS

The subjects were 25 patients who came to the hospital with hip complaints. The patients are 17 female and three male with mean age (54.5 years), ranging from 18-78 years. The demographic data, such as gender, height and weight are shown in Table **1**. The number of patients was determined by a preliminary trial (10 patients were enrolled in the trial).

Furthermore, they all gave informed consent, and the hospital's institutional review board (please see Institutional Review Board Statement and Informed Consent Statement) ethically approved the study.

**Fig. (2):**
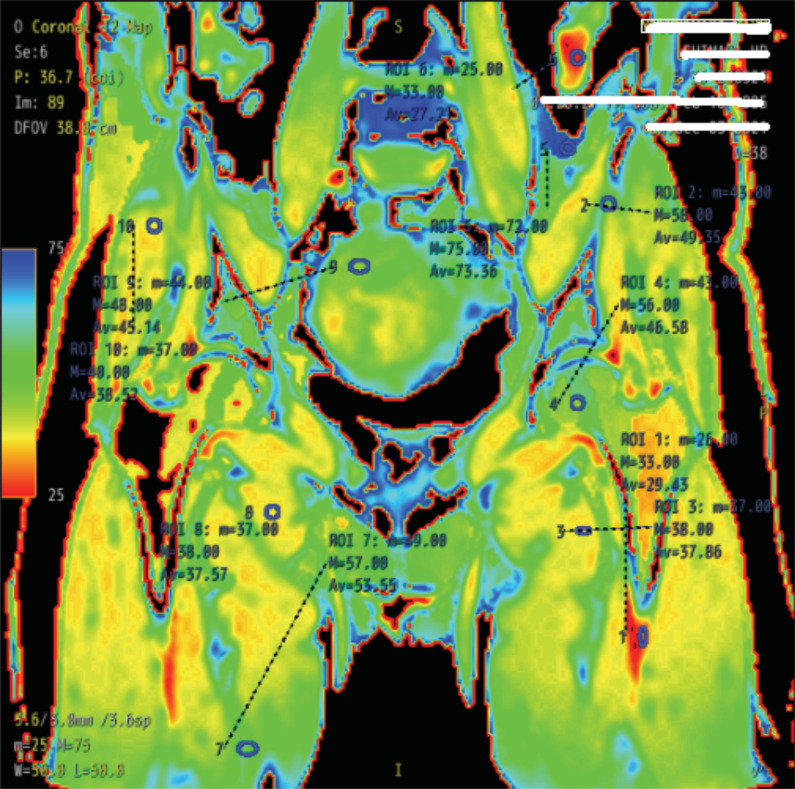
Color mapping results of a representative patient with ten ROIs to measure the T2 value.

The MRI of the hip was performed on a Signa Pioneer 3 T MR scanner (GE Healthcare Japan, Tokyo, Japan) using a flexible surface coil. First, patients were positioned supine with the hip in a neutral position. Then, the area of the quantitative MRI was taken, comprising the entire hip joint from anterior to posterior in the coronal plane, from the superior border of the pelvis to 5 cm below the lesser trochanter of the proximal femur. The two-dimensional spin-echo images were obtained using the following parameters: repetition time/echo time: 800 ms/6.61, 13.2, 19.82, 26.43, 33.04, 39.65, 46.26, 52.86 ms; field of view: 16 cm; matrix: 256 × 256, and while each slice thickness was 3 mm, the slice interval was 4.5 mm. Subsequently, the T2 color mapping was created by “Cartigram” [11,12], specific MRI software for T2 mapping in the MRI scanner, which automatically created the T2 color map (GE Healthcare, Waukesha, WI, USA). With the software, one slice was selected from each patient image. Then, the smallest range of ellipses that could be judged to comprise a single color on that image was designated as an ROI (region of interest). As a result, 10 ROIs were randomly created on the same slice. While creating the ROIs, T2 values (mean, maximum, and minimum values) were automatically calculated in the software (Fig. **2**), after which these data were recorded.

The image data of the selected slices were exported to CD-R media (a digital optical disk storage format). Then, after the image data were imported to a personal computer via CD-R, T2 values were measured from the red, green, and blue (RGB) values of the center locations of the 10 ROIs specified in the color image using our proposed method.

**Fig. (3):**
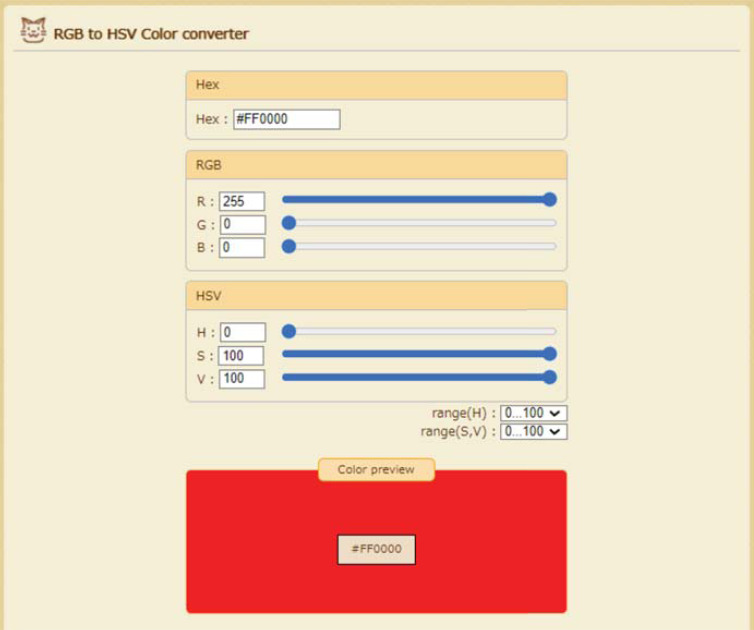
The RGB to HSV color converter on the website shows 0 of the hue value from RGB values (255,0,0).

First, RGB values were obtained using an open-source image viewing platform (WEASIS, https://nroduit.github.io/en/) [13]. When the cursor on the screen in the viewer was moved, the RGB values were displayed at any point. The values were recorded. Thereafter, HSV values were calculated on a website that converts RGB to HSV values (PEKO-STEP, https://www.peko-step.com/en/tool/hsvrgb_en.html) (Fig. **3**). There were two options: 0–360 and 0–100, based on the hue value; the latter was used in this study.

Using the above method, one author measured the T2 value in a blind fashion for the results by Cartigram [14]. Then, the measurements of the T2 value were taken by a research assistant to calculate observer errors. As a result, 250 measurements (25 cases, 10 measurements per case) were performed. The difference between the T2 values calculated by Cartigram and our proposed method was investigated by the Mann-Whitney U test, and the Pearson correlation coefficient between the two methods was also determined. Finally, the differences and the correlation coefficient were evaluated to determine whether the values were statistically significant. In addition, we investigated the difference between the T2 values calculated by Cartigram and our proposed method was related to the demographic data of the patients with the above two statistical tests (Pearson Correlation is for age, height, weight and BMI, Mann Whitney U test is for gender).

## 3 RESULTS

**Fig. (4):**
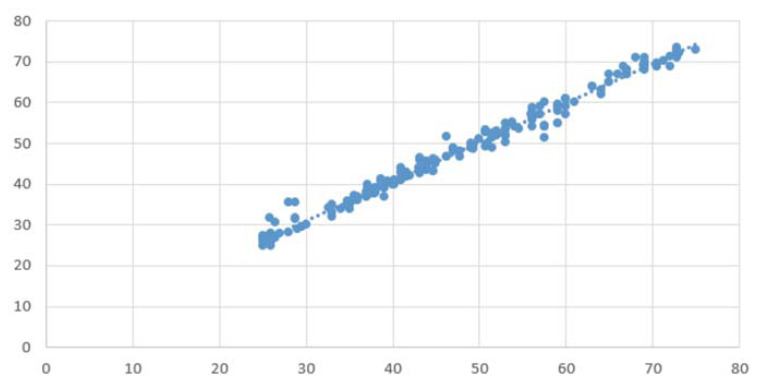
The scatter plot of the T2 values of all subjects between using dedicated software (Cartigram) on the console of the MRI scanner (x coordinate) and our method (y coordinate).

All 25 patients were measured. The mean hue value was 29.1 (SD; 17, max 66, and min 0) and the T2 value was 47.1 (SD; 13, max 75, and min 25). The mean difference (absolute difference) between the T2 values calculated using Cartigram and our method was −0.42 (1.64). Although it was statistically significant (p = 0.00003), the difference was very small. Furthermore, the correlation coefficient between the two methods was 0.9924 (Fig. **4**, p < 0.0001). The correlation coefficient range between the two methods per case ranged from 0.980 to 0.999. The intra- and inter-observer errors were 0.997 and 0.998, respectively. The demographic data of the patients were not significantly related to the difference.

## 4 DISCUSSION

Coloring on the grayscale of the medical image was introduced in the 1990s, as the color Doppler of ultrasound [15-17]. Then, positron emission tomography (PET) [18], and elastography have been widely introduced [19] as color scale or mapping. Color mapping by quantitative MRI, such as T2, T1 and T1 rho mapping, was introduced around 2000 [20-22]. So far, many other color mappings in MRI have been introduced (please see the literature review section).

Actual quantitative MRI values could only be analyzed using commercially available products, those available on the internet, or those developed independently [9, 14, 23, 24]. Cartigram for the current study is one of them, and is only conducted on the console of the MRI scanner [14]. These systems are useful for research purposes because quantitative MRI values can be shown at any point or area. Nevertheless, only processed color mapping data without knowing the numerical T2 values are provided to end users like the orthopedic physicians in an outpatient clinic, which explain the tissue conditions to the patients.

Therefore, this study proposed a method to calculate T2 values by converting RGB values on the color mapping to HSV values, obtaining hue values and evaluating their validity. Although the difference in the T2 values between using Cartigram and our method was statistically significant, it is very minimal and the correlation coefficient of the two values (0.9924) was very good. Thus, we believe that our proposed method can be validated to show the T2 value from the only color information with the color scale bar of “Jet”.

There have been several previous reports that focused on the hue value of medical images [25-27]. However, none directly focused on calculating the T2 value with the color information. Although the process of the proposed method is simple and might be natural, we consider that if specific software based on this method is available in the near future, it will allow physicians to quickly provide more detail about their patients' descriptions in an outpatient setting. Additionally, the process is independent of the vendor companies of the MRI scanner. Therefore, we consider that the proposed method would also be useful for orthopedic and other researchers who analyze quantitative MRI to improve their performances.

There were several limitations to this study. First, the number of patients enrolled was relatively small. However, the correlation coefficient range between the two methods per case was from 0.980 to 0.999, which was not so varied. Therefore, we consider the number is enough to indicate the validity of the proposed method. Second, only one pixel was set to measure the T2 value in the proposed method. Since we consider that the same method can be applied if the area of ROI is more extensive than that of this study, we will develop software with such a function shortly. Third, only the colored area could be selected on the processed color mapping, *i.e*., the black or gray areas could not be measured. In the case of some soft tissues with short T2 relaxation time, such as the meniscus, which was smaller than the lowest level (T2 = 25), the scale change of the color bar on the color mapping or color mapping by another sequence might be necessary.

## CONCLUSION

We proposed a method to measure T2 values using the hue value converted from RGB information on the processed color map to address one issue orthopedic physicians face in their outpatient clinics. Although the difference in the T2 values using dedicated software on the console of the MRI scanner and our method was statistically significant, it was small, and the correlation coefficients between the default and our proposed methods (r = 0.9924, p < 0.0001) were good. Although only one color scale bar has been investigated so far, we consider the proposed method is an alternative approach to show the T2 value with the hue value in the processed color mapping. In the near future, we are planning further study on a larger scale with our developing software.

## Figures and Tables

**Table 1:** Demographic data of the enrolled patients.

**-**	**Age**	**Gender**	**Height (cm)**	**Weight (kg)**	**BMI**
-	69	M	169	70	24.5
-	68	F	140	53	27.0
-	73	F	178	70	22.1
-	70	F	155	58	24.1
-	39	F	157	53	21.5
-	46	F	160	76	29.7
-	72	F	157	58	23.5
-	78	F	147	43	19.9
-	36	F	167	65	23.3
-	50	F	160	60	23.4
-	71	F	147	73	33.8
-	58	F	150	56	24.9
-	51	F	158	65	26.0
-	44	F	160	60	23.4
-	34	F	167	65	23.3
-	52	F	167	63	22.6
-	62	F	152	46	19.9
-	72	M	174	69	22.8
-	60	F	166	50	18.1
-	51	M	167	56	20.1
-	59	F	158	48	19.2
-	18	F	153	51	21.8
-	61	F	168	69	24.4
-	20	F	146	32	15.0
-	48	F	154	46	19.4
Ave.	54.5		159.1	58.2	23.0
S.D.*	16.3		9.4	10.6	3.8

*S.D. = standard deviation.

## Data Availability

Not applicable.

## LIST OF ABBREVIATIONS

MRI: Magnetic Resonance Image
PET: Positron Emission Tomography
RGB: Red, Green, and Blue

## STANDARDS OF REPORTING

STROBE guidelines were followed
